# Impact of the gut microbiome on immunological responses to COVID-19 vaccination in healthy controls and people living with HIV

**DOI:** 10.1038/s41522-023-00461-w

**Published:** 2023-12-20

**Authors:** Shilpa Ray, Aswathy Narayanan, Jan Vesterbacka, Ola Blennow, Puran Chen, Yu Gao, Giorgio Gabarrini, Hans-Gustaf Ljunggren, Marcus Buggert, Lokeshwaran Manoharan, Margaret Sällberg Chen, Soo Aleman, Anders Sönnerborg, Piotr Nowak

**Affiliations:** 1 Department of Medicine Huddinge, Division of Infectious Diseases, Karolinska Institutet, Stockholm, Sweden; 2 Department of Infectious Diseases, Karolinska University Hospital, Stockholm, Sweden; 3 Department of Medicine Huddinge, Center for Infectious Medicine, Karolinska Institutet, Stockholm, Sweden; 4 Department of Dental Medicine, Karolinska Institutet, Stockholm, Sweden; 5 National Bioinformatics Infrastructure Sweden (NBIS), SciLifeLab, Department of Laboratory Medicine, Lund University, Lund, Sweden; 6 Department of Laboratory Medicine, Division of Clinical Microbiology, ANA Futura, Karolinska Institutet, Stockholm, 141 52 Sweden

**Keywords:** Microbiome, Bacteriology

## Abstract

Although mRNA SARS-CoV-2 vaccines are generally safe and effective, in certain immunocompromised individuals they can elicit poor immunogenic responses. Among these individuals, people living with HIV (PLWH) have poor immunogenicity to several oral and parenteral vaccines. As the gut microbiome is known to affect vaccine immunogenicity, we investigated whether baseline gut microbiota predicts immune responses to the BNT162b2 mRNA SARS-CoV-2 vaccine in healthy controls and PLWH after two doses of BNT162b2. Individuals with high spike IgG titers and high spike-specific CD4^+^ T-cell responses against SARS-CoV-2 showed low α-diversity in the gut. Here, we investigated and presented initial evidence that the gut microbial composition influences the response to BNT162b2 in PLWH. From our predictive models, *Bifidobacterium* and *Faecalibacterium* appeared to be microbial markers of individuals with higher spike IgG titers, while *Cloacibacillus* was associated with low spike IgG titers. We therefore propose that microbiome modulation could optimize immunogenicity of SARS-CoV-2 mRNA vaccines.

## Introduction

The COVID-19 pandemic has caused more than 6 million deaths^1^. However, vaccines against SARS-CoV-2 have changed the course of the pandemic by reducing the lethality of the disease^2,3^ and the incidence of the post-acute COVID-19 syndrome^4^. Two messenger RNA (mRNA) vaccines (BNT162b2 from Pfizer-BioNTech and mRNA-1273 from Moderna) have reached an efficacy of upto 95% with minimal side effects in initial randomized clinical trials^2,5^. However, the vaccines do not fully protect against reinfection, especially with new variants^6^, with boosters being essential for enhancing host immunity. Therefore, it is important to understand the factors influencing the immunogenicity of SARS-CoV-2 vaccines, including their capacity for long-term protection against disease severity and death. Several known factors are essential for the vaccine response, such as age, medications, disease-associated comorbidities, disorders of the immune system, and inflammatory conditions^7,8^. An additional factor reported to regulate immunogenicity for several other oral and parenteral vaccines is the gut microbiota^9^. Without any bacterial stimulation, in fact, the mucosal immune system remains poorly developed, both anatomically and functionally^10^. The crosstalk between the microbial communities and immune system is crucial in maintaining homeostasis and their mutualistic relationship. In fact, gut dysbiosis has also been known to be associated with immunological disequilibrium^11^, which is often described as Th2 cell activation or T-regulatory cell deficiency^11,12^. Recent animal studies have also shed new light on the association between the gut microbiome and the immune system. In vitro studies have in fact reported the role of *Bifidobacterium adolescentis* in reducing the adhesion of Norovirus to Caco-2 and HT-29 cells^13^, two colon epithelial cell lines. *Bifidobacterium* has been shown to have broad immunomodulatory effects in humans. For example, the cellular immunity in the elderly has been reported to be augmented by *Bifidobacterium*^14,15^. In addition, *Bifidobacterium animalis* has been shown to increase NK cell activity and polymorphonuclear cell functionality in old-aged individuals. In addition, the *Clostridium* cluster XIVa and IV were shown to stimulate TGF-β for T_reg_ cell activation^16^. In fact, butyrate, a fatty acid of which Clostridia are known key producers, is pivotal in activating T_reg_ cells and their anti-inflammatory functionalities^17,18^. Interestingly, many animal and clinical studies have shown the role of the gut microbiome in vaccine immunogenicity^19,20^, such as the case of *Bifidobacterium longum*, which has been associated with antigen-specific T-cell immunity against tuberculosis and polio vaccines^21^. Actinobacteria, the bacterial phylum to which *Bifidobacterium* belongs, in fact, has been shown to have a positive correlation with adaptive immunity to certain oral (i.e., polio) and systemic vaccines (i.e., BCG, tetanus toxoid, hepatitis B virus) in infants from Bangladesh^21^. Another study has correlated the gut microbiome in infants with immune responses to the rotavirus vaccine^22,23^. Overall, the general mechanism with which the gut microbiome influences vaccine efficacy primarily involves its ability to use bacterial-derived natural adjuvants to activate specific immune pathways responsible for both innate and adaptive immune responses^24^. One such case is the expression of flagellin or LPS by the bacterial phylum Proteobacteria, leading to the activation of pattern-recognition receptors (PRRs) found on the antigen-presenting cells^19^. The concept of “endogenous adjuvant potential” was further highlighted in a study where an inactivated influenza vaccine elicited lower antibody responses in germ-free or antibiotic-treated mice^25,26^. Treating these mice with flagellated *Escherichia coli* restored the normal antibody titers. Similar results were also obtained from analogously adjuvanted vaccines against poliovirus^26^. In addition, activation of Nucleotide-binding oligomerization domain-containing protein 2 (Nod2) by gut commensals was observed to stimulate cholera toxin-mediated antigen-specific immune responses through high levels of IL-1β during oral vaccination^27,28^. Other studies have shown the immunomodulatory property of bacterial flagellin, which further activated TLR5 for improved antibody production^24,26^.

Notably, differences in gut microbiome profiles have also been associated with health status, metabolic abnormalities, and aging, which are key elements in influencing vaccine immunogenicity^29^. Studies have clearly shown a reduced efficacy of the Pfizer/BioNTech vaccine due to aging, obesity, and other comorbidities^30–32^. Elderly individuals with underlying comorbidities are at utmost risk of COVID-19 infections and display low immunogenicity against COVID-19 vaccinations, since aging leads to reduced immunity and chronic inflammation. A study compared BNT162b2 vaccine immunogenicity in two groups of patients aged <60 and >80 years, where the latter showed significantly lower spike antibody titers^33^.

Aside from the elderly, another COVID-19 risk group comprises individuals with underlying chronic diseases or disorders, which also affect the immunogenic responses to vaccines. Among them, immunocompromised individuals with primary immunodeficiencies or secondary immunodeficiencies such as HIV, are particularly prone to SARS-CoV-2 infections and display less immunogenicity to COVID-19 vaccines. Considering the significantly lower level of COVID-19 vaccine immunogenicity in immunocompromised individuals, such as people living with HIV (PLWH)^34–36^, we hypothesized that the gut microbiome might influence the efficacy of COVID-19 vaccines in these individuals. Elucidating the potential role that the gut microbiome plays in COVID-19 vaccine immunogenicity might thus offer the possibility to identify poor vaccine responders, as well as to develop potential microbiome-targeting therapies that may enhance vaccine responses. Several clinical trials, in fact, are aiming to increase the efficacy of COVID-19 vaccines by manipulating the gut microbiome. One involves the use of 5-ALA-phosphate, which is known to maintain gut homeostasis, to increase the immunogenic responses to COVID-19 vaccines^37^. Another trial involves the use of three *Bifidobacterium* strains to increase the immunogenicity of SARS-CoV-2 vaccines and reduce the side effects in elderly diabetic patients^38^. In the present work, we addressed the association between the gut microbiome and immune responses to the BNT162b2 mRNA SARS-CoV-2 vaccine. The current study presents an association between the gut microbiome and humoral/cellular COVID-19 vaccine responses in PLWH and healthy controls (HC).

## Results

### PLWH and elderly individuals displayed less immunogenicity to the BNT162b2 vaccine

All participants included were ≥18 years of age and without any prior history of SARS-CoV-2 infection, as determined by serological testing. PLWH were on antiretroviral therapy for an average of 10.8 years (33–66 years). Their median CD4^+^ T-cell count was 615 cells/mL and 86% of PLWH had less than 50 copies/mL of HIV RNA. There were no significant differences in age, BMI, or number of comorbidities between PLWH and HC (Table 1).

**Table 1:** Baseline demographic and clinical characteristics of the study participants.

	HC (*n* = 75)	PLWH (*n* = 68)	Overall (*n* = 143)	*p* value
Sex, *n* (%)				
Man	32 (43)	40 (59)	72 (50)	0.066
Woman	43 (57)	28 (41)	71 (50)	
Age (years)	52 (43.5–63)	54 (33–65.75)	54 (38–64)	0.36
BMI (kg/m^2^)	25.1 (22.6–29.2)	25 (23–27)	25 (23–28.3)	0.49
Total IgG	10.9 (9.66–12.8)	12.45 (10.77–14.5)	11.7 (9.895–13.575)	0.003
Lymphocytes (mL)	1.75 (1.5–2.2)	1.8 (1.4–2.3)	1.8 (1.5–2.3)	0.59
Creatinine (µmol/L)	72 (63–80)	84 (68–99.3)	75 (65–86)	0.0004
Ethnicity				
Caucasian	65 (87)	40 (59)	105 (73)	
Latin	1 (1)	2 (3)	3 (2)	
Asian	3 (4)	13 (19)	16 (11)	
Black	1 (1)	11 (16)	12 (9)	
Other/unknown	5 (7)	2 (3)	7 (5)	
Diet				
Omnivorous	66 (88)	63 (92)	129 (90)	
Vegetarian	6 (8)	4 (6)	10 (7)	
Others/unknown	3 (4)	1 (2)	4 (3)	
Individuals with comorbidities^a^	22 (29%)	24 (35%)	46 (32%)	0.48
Duration of ART (years)	NA	9 (4–16)		
CD4^+^ T cell count	NA	615 (290–730)		
CD4/CD8 ratio	NA	0.95 (0.47–1.35)		
CD4 nadir	NA	270 (117–437.5)		

Mann–Whitney *U* test was applied to compare the continuous variables and Fisher’s exact test to analyze the categorical variables. *P* value < 0.05 was considered significant. All baseline characteristics are illustrated as median (interquartile range) and demographic characters are illustrated as *n* (%).

*BMI* body mass index, *ART* antiretroviral treatment.

^a^Comorbidities included diabetes, hypertension, cardiovascular diseases and hypercholesterolemia.

**Fig. 1:**
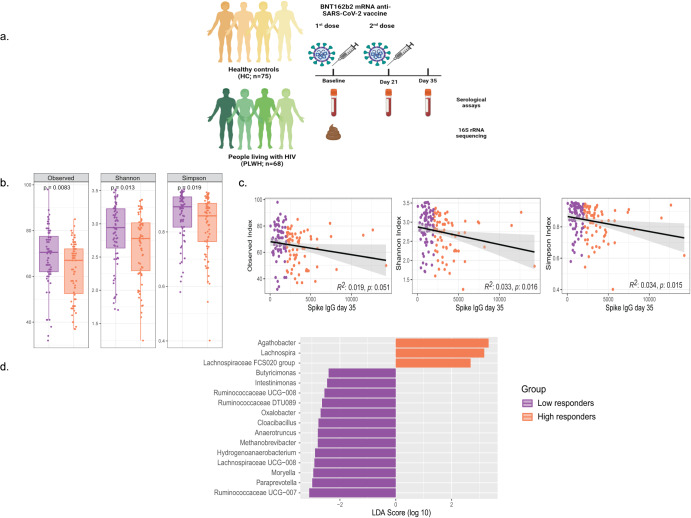
Changes in alpha diversity and bacterial genus from fecal samples collected at the baseline, to predict spike IgG titers in individuals. **a** Study design. **b** Alpha diversity decreased significantly in individuals with high spike IgG titers in the whole cohort (*n* = 143). *P* values were given by paired Mann–Whitney *U* test. **c** Linear regression relationship between alpha diversity values and spike IgG levels in all the individuals. **d** Differentially abundant bacterial genus between individuals with high and low antibodies as detected by LEfSe. LEfSe Linear Discriminant Analysis Effect Size.

Two weeks after the second mRNA vaccine dose (day 35) (Fig. 1a), the HC showed a significantly higher spike IgG titers as compared to the PLWH (*p* = 0.0001) (Supplementary Fig. 1a). Gender, BMI, and baseline total IgG levels did not affect the spike antibody titers in the whole cohort (Supplementary Fig. 1b–d). Furthermore, we observed significantly higher antibody titers in younger individuals (18–39 years) compared with middle-aged (40–59 years; *p* = 0.003) and older participants (>60 years; *p* < 0.0001) (Supplementary Fig. 1e). Age was therefore negatively correlated with spike IgG (*p* = 0.04). In addition, in PLWH, spike IgG levels were not affected by the CD4^+^ T-cell counts or CD4/CD8 ratio (Supplementary Fig. 1f, g).

### Bacterial diversity is lower in individuals with higher spike IgG titers

To better discriminate the different levels of vaccine immunogenicity among the whole cohort, individuals from both PLWH and HC group were divided into high and low responders, based on the median spike IgG titer (1972 U/mL). Notably, high responders displayed significantly lower bacterial α-diversity (richness and evenness) than low responders (Fig. 1b). All the α-diversity indices negatively correlated with spike IgG titers for the whole cohort (Fig. 1c, Observed *p* = 0.05, Shannon *p* = 0.016, Simpson *p* = 0.01). In addition, the phylogenetic diversity in high responders was reduced compared with low responders (Faith’s PD, *p* = 0.02). However, there were no major changes in β-diversity shifts in the whole cohort (Supplementary Fig. 2a), while NMDS2 scores differed significantly between high and low responders in the HC group (Supplementary Fig. 2b, *p* = 0.0002). However, no significant cluster differences were observed in the PLWH (Supplementary Fig. 2c).

In addition to bacterial diversity, we also observed significant changes in the microbiota composition between individuals with high and low spike IgG titers. A total of 258 bacterial genera were detected in the whole cohort. At the genus level, *Agathobacter* (*p* = 0.02), *Lachnopsira* (*p* = 0.03), and Lachnospiraceae FCS020 group (*p* = 0.03) were markers for high responders, according to linear discriminant analysis (LDA) scores (LDA > 2; *p* < 0.05) (Fig. 1d). *Butyricimonas* (*p* = 0.02), *Cloacibacillus* (*p* = 0.009), *Intestinimonas* (*p* = 0.02), Ruminococcaceae DTU089 (*p* = 0.006), and *Paraprevotella* (*p* = 0.02) were significantly enriched in low responders (LDA > 2; *p* < 0.05) (Fig. 1d).

**Fig. 2:**
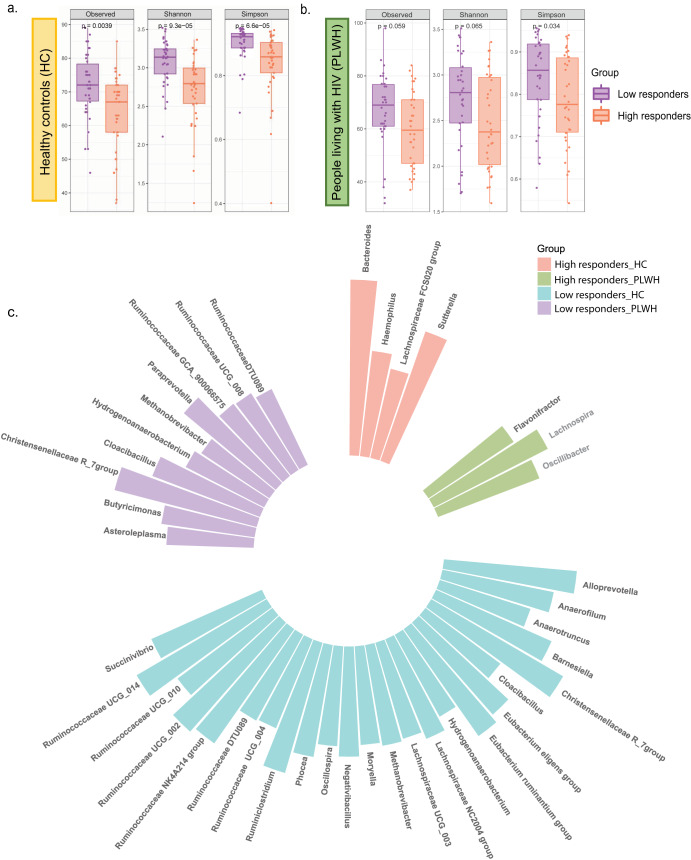
Differences in diversity and composition between high and low responders in healthy controls (HC) and people living with HIV (PLWH). **a** Alpha diversity indices between the high and low responders in the healthy controls (HC). **b** α-diversity and richness between the high and low responders in the people living with HIV (PLWH). **c** Circular plot depicting the significant microbial biomarkers abundant in either the high responders or low responders in both the HC and PLWH group via LEfSe analysis with pairwise comparison.

Likewise, negative associations between α-diversity and antibody titers were observed within the HC (Supplementary Fig. 3a) and the PLWH (Supplementary Fig. 3b) when individuals from these groups were divided into high and low responders. High responders with HC (Observed *p* = 0.004, Shannon and Simpson *p* < 0.0001) and PLWH (Simpson *p* = 0.034) displayed significantly reduced bacterial diversity than low responders within the respective groups (Fig. 2a, b). Enrichment of Bacteroidetes and depletion of Firmicutes were observed in the higher responders of the HC (*p* < 0.01). In addition, higher populations of *Bacteroides* (*p* < 0.05), *Sutterella* (*p* < 0.05), Lachnospiraceae FCS020 group (*p* < 0.05), and reduced numbers of *Alloprevotella* (*p* < 0.05), *Anaerofilum* (*p* < 0.05)*, Succinivibrio* (*p* < 0.05)*, Moryella* (*p* < 0.01)*, Negativibacillus* (*p* < 0.05), and certain members of the Ruminococcaceae family at the genus level (*p* < 0.05) were observed in the high responders within the HC (LDA > 2.3) (Fig. 2c). Within the PLWH group, on the other hand, *Flavonifractor*, *Lachnospira*, and *Oscillibacter* were increased in high responders (*p* < 0.05, LDA > 2.6), whereas *Butyricimonas* and *Paraprevotella* were depleted in low responders (*p* < 0.05, LDA > 2) (Fig. 2c). Both the HC and the PLWH groups displayed an increased abundance of *Hydrogenoanaerobacterium*, *Methanobrevibacter*, *Cloacibacillus*, and Ruminococcaceae DTU089 in individuals with low spike IgG titers.

### Spike-specific CD4^+^ T-cell response shows negative association with α-diversity

**Fig. 3:**
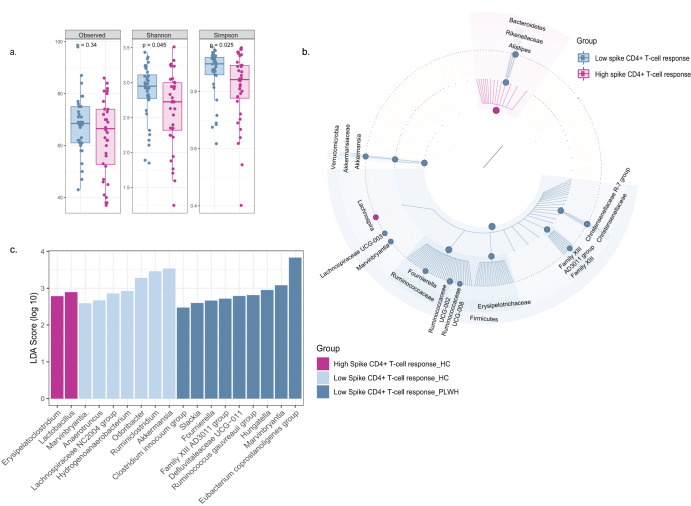
Changes in microbial diversity and composition affected by spike-specific CD4^+^ T-cell response. **a** Alpha diversity changes between individuals with high and low spike-specific CD4^+^ T-cell response (*n* = 90). **b** GraPhlAn plot depicting the significant changes in bacterial populations in the individuals with high or low levels of CD4^+^ T-cell response. **c** Differences in the richness of bacterial genus between the people with high and low CD4^+^ T-cell responses to spike protein within the HC and PLWH group via LEfSe analysis.

CD4^+^ T-cell response to the spike protein of SARS-CoV-2 was evaluated in the HC (*n* = 45) and PLWH (*n* = 45) at day 35. We stratified all individuals (*n* = 90) into two groups based on the magnitude of their spike CD4^+^ T-cell response (median 0.36%). A significant decline in the α-diversity was found in individuals with higher levels of spike-specific CD4^+^ T-cells (Fig. 3a) as compared to individuals with low levels of spike-specific CD4^+^ T-cells (Shannon *p* = 0.045, Simpson *p* = 0.025). Similar to spike IgG titers, we also found a negative association between α-diversity and spike-specific CD4^+^ T-cell response (observed *p* = 0.003, Shannon *p* = 0.001, Simpson *p* = 0.005) from linear regression analysis (Supplementary Fig. 4). There were shifts in the β-diversity (*p* = 0.01) with unique clustering patterns specific to each group (Supplementary Fig. 5). In addition, individuals with low spike-specific CD4^+^ T cell response had more Firmicutes (*p* = 0.005, LDA > 4.5) and less Bacteroidetes (*p* = 0.005, LDA > 4) compared with high responders (Fig. 3b). Moreover, Ruminococcaceae (*p* = 0.02, LDA > 4.5), Erysipelotrichaceae (*p* = 0.04, LDA > 3.5), and Akkermansiaceae (*p* = 0.03, LDA > 3.5) were enriched in individuals with low CD4^+^ T cell responses (Fig. 3b). Individuals eliciting a higher magnitude of CD4^+^ T cell response had an increased abundance of *Lachnospira* (*p* = 0.035, LDA > 3). In contrast, the low responders showed an increased abundance of *Akkermansia* (*p* = 0.035, LDA > 3), *Fournierella* (*p* = 0.014, LDA > 3), and *Alistipes* (*p* = 0.029, LDA > 3.5) (Fig. 3b).

Similarly to the whole cohort, a consistent negative association between spike-specific CD4^+^ T cell response and α-diversity was also observed within the HC (Supplementary Fig. 6a, b) and PLWH (Supplementary Fig. 6c, d). The low responders in the PLWH were enriched with Firmicutes (*p* = 0.01, LDA > 4.5) and depleted of Bacteroidetes (*p* = 0.046, LDA > 4.5). Erysipelotrichaceae (*p* = 0.01), Eggerthellaceae (*p* = 0.006), and Succinivibrionaceae (*p* = 0.036) were detected as signatures of low responders. Both the HC and PLWH had a high abundance of *Marvinbryantia* in communities with low CD4^+^ T cell response (*p* < 0.05). Higher numbers of *Fournierella* (*p* = 0.03) were identified in low responders within the PLWH (Fig. 3c). On the other hand, the high responders in the HC showed a higher abundance of Lactobacillaceae (*p* = 0.01, LDA > 3) and a decline of Akkermansiaceae (*p* = 0.02, LDA > 3.5). At the genus level, the high responders in the HC displayed an enrichment of *Lactobacillus* (*p* = 0.014). Instead, *Akkermansia* (*p* = 0.017), *Ruminiclostridium* (*p* = 0.037), and *Hydrogenoanaerobacterium* (*p* = 0.047) were more abundant in low spike-specific CD4^+^ T cell responders (Fig. 3c).

Furthermore, we explored the correlation of bacterial taxa associated with spike CD4^+^ T-cell response and spike IgG titers (Supplementary Table 1). In the whole cohort, *Sutterella* (*p* = 0.01), *Bifidobacterium* (*p* = 0.015), *Bacteroides*, *Lachnospira*, and *Lactobacillus* showed possible positive correlation, while *Escherichia-Shigella* (*p* = 0.015), *Marvinbryantia* (*p* = 0.002), Ruminococcaceae DTU089 (*p* = 0.018), *Methanobrevibacter* (*p* = 0.028), and *Cloacibacillus* (*p* = 0.015) showed negative correlation with antibody levels (Supplementary Fig. 7).

### Gut microbiome diversity is affected by age

A relative decline in diversity and richness was observed in young adults (18–39 years, *n* = 37) as compared to older individuals (>60 years, *n* = 50) (Observed, *p* < 0.001 and Shannon, Simpson, *p* < 0.0001, Supplementary Fig. 8a). Moreover, there was a significant positive association between age and α-diversity (Supplementary Fig. 8b, observed *p* = 0.0002, Shannon *p* = 0.00001, Simpson *p* = 0.0001). Similarly, a significant shift in β-diversity was observed among the different age groups (*p* = 0.03) (data not shown). At the genus level, certain members of the Ruminococcaceae family (*p* < 0.05), *Butyricimonas* (*p* = 0.01), *Ruminiclostridium* (*p* = 0.005), *Hydrogenoanaerobacterium* (*p* = 0.005), *Fournierella* (*p* = 0.009), Christensenellaceae R_7 group (*p* = 0.007) and *Methanobrevibacter* (*p* = 0.007) showed increased abundance in older individuals (>60 years) (LDA > 2.5, Supplementary Fig. 8c). In young adults we observed an enrichment of *Lachnospira* (*p* = 0.02), *Bacteroides* (*p* = 0.02), and *Agathobacter* (*p* = 0.009) which were also enriched in individuals with high spike IgG titers (LDA > 3). Within the HC and PLWH groups, we observed a similar pattern of relative decrease in the α-diversity indices of young adults compared to the elderly and middle-aged (Supplementary Fig. 8d, e). Predominantly, the Bacteroidetes population was significantly enriched in younger adults as compared to the older individuals (*p* = 0.001), who harbored a higher abundance of Firmicutes (*p* = 0.006; LDA > 4.5) (Supplementary Fig. 8f). The Firmicutes/Bacteroidetes (F/B) ratio was highest in the elderly and lowest in the youths.

### Gut microbiome profiles are affected by total IgG levels at baseline

In addition, we studied whether total IgG levels at baseline were associated with specific microbiome profiles, since we observed a borderline significant association with spike IgG levels in our cohort (*p* = 0.09). Stratifying the individuals into those with high and low total IgG levels at baseline, we found a decline in the bacterial diversity of the individuals with high total IgG levels (data not shown). The most differential genera among all samples from our analysis (individuals with high and low total IgG levels) were *Anaerostipes* (*p* < 0.01), *Fournierella* (*p* < 0.01), *Mitsuokella* (*p* < 0.05), and *Lactobacillus* (*p* < 0.05) (Supplementary Fig. 9a). Interestingly, an increased abundance of *Anaerostipes* (*p* < 0.01, LDA > 3), *Bacteroides*, and *Bifidobacterium* were detected in the individuals with high levels of total IgG at baseline, and these genera were also likely to be positively correlated with spike IgG titers (Supplementary Fig. 9b, c).

### Gut microbiota α-diversity and composition are associated with spike IgG levels irrespective of age and disease status

**Fig. 4:**
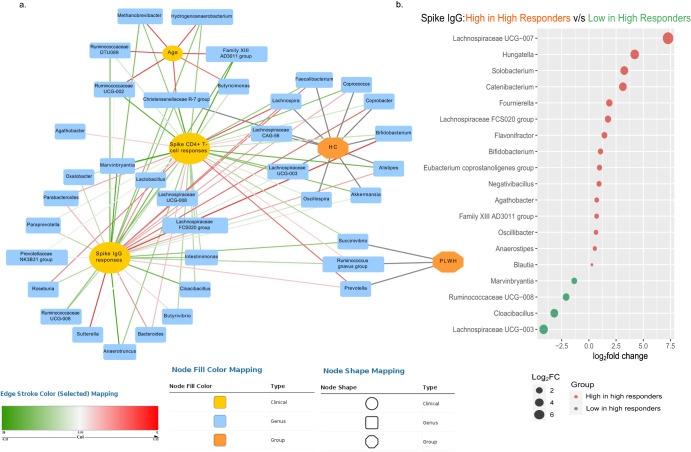
Association of gut microbiota with spike IgG levels. **a** Network analysis of linking different bacterial genus to clinical factors such as spike IgG levels on day 35, age, and spike-specific CD4^+^ T-cell responses. **b** DESeq2 analysis to observe the differential abundance through estimation of fold change of each microbial taxon associated with antibody levels, irrespective of different clinical parameters, such as age, gender, and disease group.

We further integrated the cellular and humoral immune responses on day 35, with selected baseline factors, such as age and disease group, into a network and correlated the microbial taxa, which were significantly associated with said clinical characteristics and immunological responses. Overall, from our predictive models, we observed a positive association of spike IgG levels with *Lachnospira*, *Faecalibacterium*, and *Bifidobacterium*, which were also markers of the HC group (Fig. 4a). *Agathobacter*, *Lactobacillus, Bacteroides*, and *Lachnospira* positively correlated with both spike IgG levels and spike-specific CD4^+^ T-cell responses (Fig. 4a). The microbes positively linked with age (*Hydrogenoanaerobacterium*, *Methanobrevibacter*, Ruminococcaceae DTU089, *Butyricimonas*) showed a negative correlation with both spike IgG levels and CD4^+^ T-cell responses. Spike IgG levels and CD4^+^ T-cell responses were negatively associated with *Methanobrevibacter*, Ruminococcaceae DTU089, *Paraprevotella*, *Marvinbryantia*, *Cloacibacillus*, and *Succinivibrio* (Fig. 4a).

Furthermore, we carried out a DESeq2 analysis to model the absolute abundance of each microbial taxon associated with antibody levels irrespective of other clinical parameters (such as age, gender, disease group) to determine the log_2_fold change (effect size estimate) for differential changes in microbial abundance as true absolute counts. We observed significant changes in the abundance of several gut microbes directly associated with spike IgG. Thus, individuals with high spike IgG titers revealed enrichment of *Bifidobacterium* (*p* = 0.03), *Faecalibacterium* (*p* = 0.03), *Blautia* (*p* = 0.03), *Catenibacterium* (*p* = 0.03), and *Hungatella* (*p* = 0.005) from this analysis (Fig. 4b). *Bifidobacterium* was increased in subjects with high spike IgG titers and high baseline total IgG. Conversely, individuals with low antibody titers showed a higher abundance of *Cloacibacillus* (*p* = 0.0001) (Fig. 4b).

In univariate analysis, the number of observed species, Shannon diversity, Simpson index, and age were all significantly associated with spike IgG levels (Table 2). To reveal the independent association between α-diversity and humoral response to COVID-19 vaccination, we conducted multiple regression analysis. We found a significant association of bacterial abundance (Shannon *p* = 0.02, Simpson *p* = 0.019) with spike IgG levels, independent of age (Table 2 and Supplementary Table 2). However, age showed a significant impact when the number of observed species and age were correlated to spike IgG response (*p* = 0.04). The results suggest that microbial abundance is independent of age and influences spike IgG levels. Nonetheless, the observed richness shows age dependency to affect spike IgG levels, while richness estimates are unaffected by species abundance.

**Table 2:** Univariate and multivariate regression analysis for analyzing the linear relationship between baseline characteristics and Spike IgG response among individuals.

	*p* value	*p* value^a^ (FDR-corrected)	Beta coefficient
Observed	0.05	0.09	–0.16
Shannon	0.01	0.06	–0.20
Simpson	0.01	0.06	–0.20
Age	0.03	0.09	–0.17
Gender	0.73	0.78	0.02
Total IgG	0.09	0.13	–0.14
BMI	0.78	0.78	–0.02
Observed, age	0.04	0.04	–0.76
Shannon, age	0.02	0.03	–0.04
Simpson, age	0.019	0.03	0.22

^a^Significance of difference (*p* < 0.05) expressed as false discovery rate-corrected *p* value.

Using multivariate analysis, we further analyzed the effect of other baseline clinical factors, such as gender and total IgG, and observed that the α-diversity significantly affected the spike IgG response (Supplementary Table 3, observed *p* = 0.02, Shannon *p* = 0.006, Simpson, *p* = 0.005). Gender showed no effect on antibody titers, followed by age and IgG. In addition, the α-diversity significantly impacted the spike-specific CD4^+^ T-cell responses in the cohort irrespective of age, gender, and total IgG (Supplementary Table 3, observed *p* = 0.006, Shannon *p* = 0.0009, Simpson, *p* = 0.004).

## Discussion

SARS-CoV-2 mRNA vaccines are highly immunogenic and effective in protecting from severe COVID-19^2^. However, several factors, such as increasing age, underlying medical conditions, and concomitant medications, are associated with lower vaccine efficacy^7,8,39^. Increasing evidence, from clinical cohorts, interventional studies, and animal models, show that gut microbiota plays a significant role in modulating responses to vaccination^9,40^.

In our study, we investigated whether the baseline gut microbial diversity and composition would affect the immunogenicity of mRNA BNT162b2 SARS-CoV-2 vaccine in PLWH, as immunocompromised individuals possess lower immunogenic responses to these vaccines. Interestingly, α-diversity metrics were negatively associated with spike IgG titers post-vaccination, both in the whole cohort and within the subgroups of HC and PLWH. In addition, we found that the elevated levels of spike-specific CD4^+^ T-cell responses were associated with reduced microbiome diversity, thus further validating the association of the microbiome with the immunogenicity of the BNT162b2 mRNA vaccine. These results are in line with data presented by previous studies using vaccines against other viral diseases. A collection of studies reported that similar vaccines (both oral and parenteral) elicit impaired immunogenicity in individuals living in low-income countries compared with people from high-income countries, due to their differences in gut microbiome profiles^19^. Moreover, two studies on oral polio vaccine, in India and China, showed a low bacterial diversity in individuals with high vaccine efficacy^41,42^. Furthermore, the role of microbiota in modulating T-cell responses has been shown in influenza infection^43^. Lastly, antibiotic treatment prior to H1N1 vaccination altered the microbial diversity, resulting in decreased influenza-specific IgG1 and IgA antibody titers^44^.

In addition to bacterial diversity, this study showed that the baseline microbiota composition could potentially influence the immunological effects of the SARS-CoV-2 vaccines. We observed that certain bacterial genera at the baseline were associated with vaccine immunogenicity, measured by IgG spike and spike-specific CD4^+^ T-cell responses. Interestingly, in the network analysis, *Agathobacter*, *Lactobacillus, Bacteroides*, and *Lachnospira* were positively correlated with both spike IgG levels and spike-specific CD4^+^ T-cell responses. These bacteria with known immunomodulatory properties could contribute to higher immune responses to vaccinations^45–47^. *Bacteroides*, in fact, have been shown to have a key role in vaccine responses due to the induction of homeostatic immune priming. For instance, the LPS of *Bacteroides thetaiotaomicron* acts as an adjuvant, enhancing the production of hepatitis B virus antigen-specific antibodies^48^. In addition, another study revealed the association of different *Bacteroides* strains with diverse response levels to rotavirus vaccine^49^. Overall, *Bacteroides* are resident commensals, acting as major polysaccharide degraders in the gut for butyrate production^50^. Conversely, *Methanobrevibacter*, Ruminococcaceae DTU089, *Marvinbryantia*, *Cloacibacillus*, and *Succinivibrio* were enriched in the baseline microbiome of individuals with low IgG titers and low spike-specific CD4^+^ T-cell responses. This is in line with previous reports, as *Cloacibacillus* has been previously associated with bacteremia^51^, *Succinivibrio* was found in inflammatory conditions^52^ and previous studies have reported the enrichment of *Marvinbryantia* spp., which are SCFA-producers, in the elderly^53^, who experience a lower response to vaccinations, as observed in our study. Similarly, in our study, *Marvinbryantia* was observed to be less abundant in the younger group in the whole cohort and within the HC and PLWH groups. Moreover, our findings are also consistent with results from Borgognone et al.^54^, in which lower α-diversity and gene richness were observed in PLWH, who showed better immunogenic response to an HIV vaccine. These individuals, dubbed viremic controllers, were observed to have significantly higher Bacteroidales/Clostridiales ratio and lower Methanobacteriales than the non-controllers^54^. Our study also shows similar findings in individuals with high spike IgG levels, with less abundance of bacterial groups belonging to Clostridiales and Methanobacteriales, such as Ruminococcaceae DTU089 and *Methanobrevibacter*, respectively. The abundance of certain bacterial groups belonging to Clostridiales in low responders might be potentially associated with metabolic pathways related to methanogenesis and carbohydrate synthesis.

Notably, in the present work, *Bifidobacterium* and *Faecalibacterium* showed significant association with high spike IgG titers, irrespective of other factors, from our predictive models. This is in line with earlier studies, where enrichment of *Bifidobacterium* was correlated with CD4^+^ T-cell responses and higher antibody titers to parenteral and oral vaccinations^41,55^. In addition, *B. adolescentis* was also associated with a higher rate of neutralizing antibodies after CoronaVac immunization^56^. Current clinical trials are exploring the effect of supplementation with different *Bifidobacterium* species on immune responses to SARS-CoV-2 vaccine^38^. Interestingly, our findings on microbiome associations with mRNA vaccine immunogenicity in the whole cohort were also consistent within the PLWH group. This is of note, since the gut microbial composition of PLWH is altered due to gut dysbiosis occurring during the course of HIV-1 infection and further changed during antiretroviral therapy, as shown by our previous studies^57,58^. Interestingly, in our study, we observed different bacterial groups from *Lachnospiraceae* to be associated with either high or low spike IgG levels. Generally, the bacterial members belonging to the family *Lachnospiraceae* are well-known producers of SCFAs in colon mucosa-associated microbiota. *Lachnospiracea* FSC020, which shows a positive association with spike IgG levels in our study, has been reported to have a potential positive association with the production of acetate and propionate^59^ and to be linked to circulating lipid metabolites in blood^60^. In addition, some members of Lachnospiraceae family, such as Lachnospiraceae UCG-003 can potentially protect against colon cancer by butyrate production^61^. While existing literature shows Lachnospiraceae UCG-008 to be negatively associated with age^62^ and saturated fatty acid intake^63^ and has decreased abundance in conditions like liver cirrhosis and hepatocellular carcinoma, our findings reveal the abundance of this taxon in low responders to COVID-19 vaccine^64^.

The key findings of this study link α-diversity with spike IgG responses, which in turn is also influenced by age. Although α-diversity has been known to play a crucial role in association studies, we believe that the gut bacterial composition and its functional aspects play a much more important role in influencing the landscape of gut balance. In this study, individuals with low α-diversity showed better immunogenic responses and an abundance of *Bifidobacterium*, which is known to have a protective function in the gut. In this study, we also observed younger adults with low α-diversity to have better vaccine immunogenicity compared to elderly individuals with higher α-diversity. In addition, individuals with low immunogenic responses were observed to have high α-diversity. This overall demonstrates a negative association between diversity and spike IgG levels and a positive association between age and α-diversity. Higher α-diversity in elderly individuals has also been observed in a few other studies^65–67^. Moreover, when investigating the gut composition, we observed bacterial genera specific to younger participants, such as *Lachnospira*, *Bacteroides*, and *Agathobacter* to be positively associated with spike IgG levels. Similarly to the findings from another study, we also detected enrichment of potential pathobionts in elderly individuals, which have higher α-diversity, such as an increase of Enterobacteriaceae which has been correlated with frailty in elder individuals^65^. At the genus level, we also observed some microbial signatures of pathobionts such as *Escherichia-Shigella*, and *Enterococcus* in the elderly group, as corroborated by the literature^66,67^. Similarly to other studies^65–68^, our analyses also detected in this group an abundance of certain members of the Ruminococcaceae family, *Butyricimonas*, Christensenellaceae R_7 group, *Akkermansia*, members of Erysipelotrichaceae and a reduction of *Faecalibacterium*, a pattern which has been linked to inflammatory disorders^69^. Ruminococcaceae are well-known symbionts present in the human gut generating SCFA^70^ to enhance the protective functions of the intestinal epithelium and prevent infections from opportunistic pathogens^71^. An enrichment of Ruminococcaceae diversity (Supplementary Fig. 10) might be associated with better metabolic plasticity and versatility of the gut in elderly individuals^65^. Notably, while the elderly population has been reported to have a lower F/B ratio^72^, our study shows it to display a higher F/B ratio. This has also been observed in another study cohort from Ukraine^73^. In fact, the LefSe data shows the abundance of Firmicutes in elderly and Bacteroidetes in younger individuals, both of which are the predominant bacterial phyla colonizing the gut. As a matter of fact, certain key species within the immunomodulatory Gram-negative Bacteroidetes phylum have been reported to exert anti-inflammatory effects through T-cell modulation^74^. Lastly, the elderly population showed an abundance of gut microbes, such as *Hydrogenoanaerobacterium*, *Methanobrevibacter, Negativibacillus*, which were also observed in individuals with low spike IgG levels, which is in line with the observed negative association between age and spike IgG levels in our study.

The mechanisms by which the gut microbiota modulates immunological responses to vaccines are not yet fully understood. Several potential mechanisms, however, have been anticipated. Microbiota-derived flagellin, peptidoglycan, and lipopolysaccharide can act as natural adjuvants to vaccination, recognized by PRRs^19,75^. Another important mechanism concerns gut integrity, which is crucial in regulating immune responses, and is disrupted in a state of inflammation, malnutrition, or by antibiotic treatment^76^. The gut–barrier integrity is modulated by certain bacterial metabolites, which induce the expression of tight junction proteins, thereby maintaining the epithelial integrity^77^. Microbiota-derived metabolites, such as SCFAs (acetate, butyrate, and propionate), tryptophan, and secondary bile acids can, therefore, directly and indirectly alter immune responses^19^. They provide energy sources for enterocytes, strengthen the epithelial barrier, and can act as signaling molecules in regulatory pathways of intestinal and systemic immunity. SCFAs also regulate T-cell metabolism and can augment B-cell metabolism, enhancing pathogen-specific antibody responses^78,79^. In the present study, certain butyrate producers, such as *Lachnospira*, *Catenibacterium*, and *Faecalibacterium*, were enriched in individuals with higher immunogenic responses to the BNT162b2 vaccine, and metabolites derived from these bacteria might likely have an association with higher vaccine immunogenic responses. As a critical butyrate producer, *Faecalibacterium prausnitzii* has been previously added to probiotic formulations for restoring gut health^80,81^. Overall, we hypothesize that microbiota-derived metabolites, such as butyrate, indole, and bile acids, might possibly have a strong potential to improve vaccine responses, and quantifying such metabolites could be an interesting topic for future research.

The primary limitation of the present study lies in its cross-sectional design, preventing definitive conclusions from being drawn due to the sample being collected at a single time point. Another constraint pertains to the specific sequencing approach used on the samples. Employing 16S RNA sequencing, indeed, did not yield a high resolution of the microbiome, lacking species- and strain-level information along with not being able to evaluate and understand their functional and metabolic potential. However, one of the strengths of the study is the substantial number of individuals for each of the two groups that were enrolled in the clinical study. The participants were followed using a strict protocol, where we carefully selected the individuals who did not undergo antibiotic treatment nor showed signs of prior or ongoing SARS-CoV-2 infection.

Conclusively, we observed that the baseline gut microbiota diversity and abundance significantly affected immunologic responses to SARS-CoV-2 vaccines, irrespective of age, gender, and total IgG. Although age had a significant association with both the microbial diversity and spike IgG levels, results from multiple regression analyses showed that baseline microbial abundance was independent of age and had a dominant impact on modulating antibody response to the COVID-19 vaccine.

In summary, we describe novel findings on a strong association between the intestinal microbial diversity, composition, and immunogenicity of a mRNA SARS-CoV-2 vaccine. These findings may influence the development of microbiota-centered therapies to optimize immunogenicity and durability of vaccination. For example, the augmentation of vaccination with concomitant treatment with probiotics, prebiotics, or diet could be a scalable intervention for individuals with lower vaccine responses, like elderly or immunocompromised individuals, such as PLWH. The feasibility of such therapies is considerable, as a systematic review of results from 26 interventional studies in humans using probiotics to enhance the efficacy of 17 different vaccines revealed positive outcomes in half of the trials^82^. Furthermore, a recent preclinical study showed the effectiveness of synbiotics in enhancing immunogenicity of the cholera vaccine in mice that were colonized with a poorly immunogenic infant’s microbiota^83^. In addition, the presented microbiome association with the vaccination outcome should warrant more careful use of treatments that influence the gut microbiome, like antibiotics^76^. Overall, these results pave the way for future studies, which could lead to a deeper understanding of microbiota modulation of vaccine responses in different populations and disease conditions.

## Methods

### SARS-CoV-2 COVAXID vaccine cohort and sample collection

An open-label, non-randomized clinical trial was initiated during spring 2021 at the Karolinska University Hospital, Stockholm, Sweden, to investigate the safety and clinical efficacy of the mRNA BNT162b2 vaccine (Comirnaty®, Pfizer/BioNTech) in healthy controls and immunocompromised patients (EudraCT no. 2021-000175-37, clinicaltrials.gov no. 2021-000175-37). The ethical permit was granted by the Swedish Ethical Review Authority (ID 2021-00451), and all participants provided written informed consent (Table 1)^84^. The study cohort of that study included individuals belonging to six groups^84^; however, the current study focused on two of these groups, PLWH (*n* = 90) and healthy controls (HC) (*n* = 90). Figure 1a displays the study design, with the fecal samples from the PLWH and HC group being collected at baseline for DNA extraction before they had received two doses of the mRNA vaccine three weeks apart. The extracted DNA was further sent for 16S rRNA sequencing. Humoral and cellular responses to SARS-CoV-2 vaccination were evaluated on day 35 after the 1st vaccine dose. Subjects with detectable baseline spike antibodies against SARS-CoV-2, antibiotic treatment (3 months before vaccination), and those with missing spike IgG data at day 35 were excluded from further analysis (PLWH: *n* = 22; HC: *n* = 15). The fecal samples were collected in RNA/DNA shield (Stratec, Germany). DNA was extracted using ZymoBIOMICS™ DNA Kit (Zymo Research, USA), according to the manufacturer’s specifications, and sequencing analysis was performed on the MiSeq platform.

### Detection of spike IgG and spike CD4^+^ T-cell responses

Blood samples were collected at baseline and on day 35. Elecsys® Anti-SARS-CoV-2 S RBD (Roche Diagnostics) was used to identify and quantify antibodies specific to SARS-CoV-2 spike protein in serum samples^84^. Spike-specific CD4^+^ T-cell responses were quantified using activation-induced marker assays via up-regulation of CD69 and CD40L (CD154), as previously described^85^. Total IgG levels were analyzed by routine diagnostic methods with the Roche Elecsys anti-SARS-CoV-2 S enzyme immunoassay, at the Clinical Immunology laboratory of Karolinska University Hospital^84^. For PLWH, CD4^+^ and CD8^+^ T-cell counts and HIV viral load (VL) were determined by flow cytometry and Cobas Amplicor (Roche Molecular Systems Inc., USA), respectively^86^.

### Microbiome analysis

Paired-end Illumina reads were checked for quality using FastQC^87^ and trimmed using Cutadapt^88^. During the pre-processing step, primers, adapters, and low-quality (*Q* < 30) reads were removed. The taxonomic classification and analysis of the trimmed reads were performed using dada2^89^ within Qiime2^90^ in combination with SILVA database (SILVA v132)^91^. DADA2 was used for denoising, read pair merging and PCR chimera removal which reduces sequence errors and dereplicates sequences (Supplementary Table 4). α-diversity of the samples was estimated using the R function *estimate_richness* in R package phyloseq (v1.30.0)^92^ and visualized using R package ggplot2 (v3.3.5)^93^. The diversity indices such as Observed, Shannon, and Simpson were performed to calculate the richness and diversity of the samples. The distances between the samples were clustered based on the Bray-Curtis distance metrics and visualized using non-metric multidimensional scaling (NMDS) ordination plots, and the significance of the different factors on the beta-diversity was calculated based on PERMANOVA using the vegan package (v2.5.7) (Adonis function). The relative abundance of the samples was calculated using Qiime2. Linear discriminant analysis Effect Size (LEfSe) was employed to determine the significant microbial communities between the groups^94^. All the barplots were visualized using the R package ggplot2. LefSe plot of spike CD4^+^ T-cell response and specific microbial biomarkers were visualized using GraPhlAn^95^. Multivariate regression analysis was used to predict the factors related to spike IgG. α-diversity indices, age, gender, total baseline IgG, and BMI were used as covariates in the regression analysis (R package). The models were predicted using stepwise backward selection, and *p* values < 0.05 were considered significant. Furthermore, we performed multiple hypothesis testing using a false discovery rate (FDR). Multivariate analysis of variance (MANOVA) was used to access the different patterns between the multiple dependent variables simultaneously and it was performed using the R function manova(). The Kruskal–Wallis test was performed for the post hoc analysis for MANOVA. The OTUs belonging to Ruminococcaceae were segregated to assess the richness and diversity of Ruminococcaceae family between elderly and younger individuals (Supplementary Fig. 10).

### Correlation and network analysis

Correlation analyses were based on the Spearman correlation method using R package psych (v2.2.3)^96,97^. Benjamini–Hochberg method was used to adjust the *p* values for the multiple testing. The results were visualized using the R package corrplot (v0.92)^98^. The input variables for the networks were bacterial genus, age, spike CD4^+^ T-cell response, and the antibody level at day 35, visualized using Cytoscape (v3.6.1)^99^. Differential abundance analysis between high responders and low responders was performed using R package DESeq2 (v1.26.0)^100^ and visualized using bubble plots. Bacterial taxa with *p* values less than 0.05 were considered significant.

### Reporting summary

Further information on research design is available in the Nature Research Reporting Summary linked to this article.

### Supplementary information

Supplementary figures

Supplementary tables

Reporting Summary

## Data Availability

The metadata and raw 16S rRNA gene sequence data generated and analyzed during this study are deposited at the NCBI SRA database (Project number: PRJNA902956).

## Code availability

All the codes are available at GitHub: https://github.com/Asw614/Gut-microbiome-on-immunological-responses-to-COVID-vaccination.git.
