# Supplementary material for: Impact of the gut microbiome on immunological responses to COVID-19 vaccination in healthy controls and people living with HIV
Source: NPJ Biofilms Microbiomes. 2023 Dec 20;9:104. doi: 10.1038/s41522-023-00461-w (PMC10733305; doi:10.1038/s41522-023-00461-w)
Supplement: Supplementary file 1 — Supplementary figures [file 41522_2023_461_MOESM1_ESM.pdf]

Supplementary Figures

Impact of the gut microbiome on immunological responses to COVID-19 vaccination in healthy controls and people living with HIV

Shilpa Ray[1*], Aswathy Narayanan[1], Jan Vesterbacka[1,2], Ola Blennow[1,2], Puran Chen[4], Yu Gao[4], Giorgio Gabarrini[3], Hans-Gustaf Ljunggren[4], Marcus Buggert[4], Lokeshwaran Manoharan[5], Margaret Sällberg Chen[3], Soo Aleman[1,2], Anders Sönnerborg[1,2,6,] and Piotr Nowak[1,2]

[1] Department of Medicine Huddinge, Division of Infectious Diseases, Karolinska Institutet, Stockholm, Sweden
[2] Department of Infectious Diseases, Karolinska University Hospital, Stockholm, Sweden
[3] Department of Dental Medicine, Karolinska Institutet, Stockholm, Sweden
[4] Department of Medicine Huddinge, Center for Infectious Medicine, Karolinska Institutet, Stockholm, Sweden
[5] National Bioinformatics Infrastructure Sweden (NBIS), SciLifeLab, Department of Laboratory Medicine, Lund University, Lund, Sweden
[6] Department of Laboratory Medicine, Division of Clinical Microbiology, ANA Futura, Karolinska Institutet, Stockholm 141 52 Sweden

Running title: Gut microbiome predicts COVID-19 vaccine response.

***Corresponding author:** Shilpa Ray (shilpa.ray@ki.se)

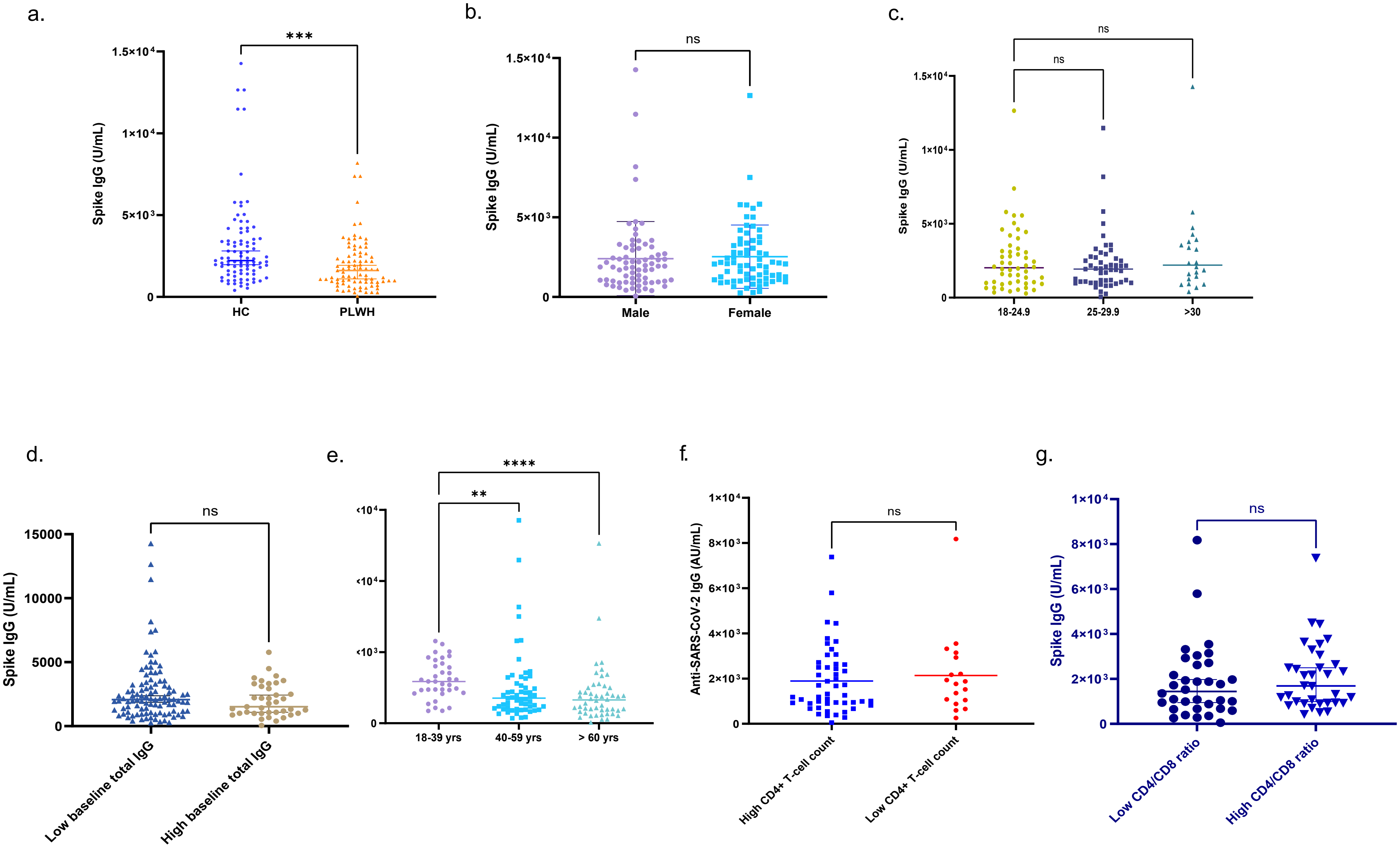

Supplementary Figure 1: Changes in the spike IgG levels between (a) Healthy controls (HC) and people living with HIV (PLWH), (b) Males and female participants of the cohort, (c) Individuals with different BMI, 18-24.9, 25-29.9 and >30. (d) Individuals with high and low total IgG levels at baseline, (e) Individuals in different age groups, 18-39, 40-59 and >60 years, (f) PLWH with high and low actual $CD4^+$ count and (g) high and low CD4/CD8 ratio.

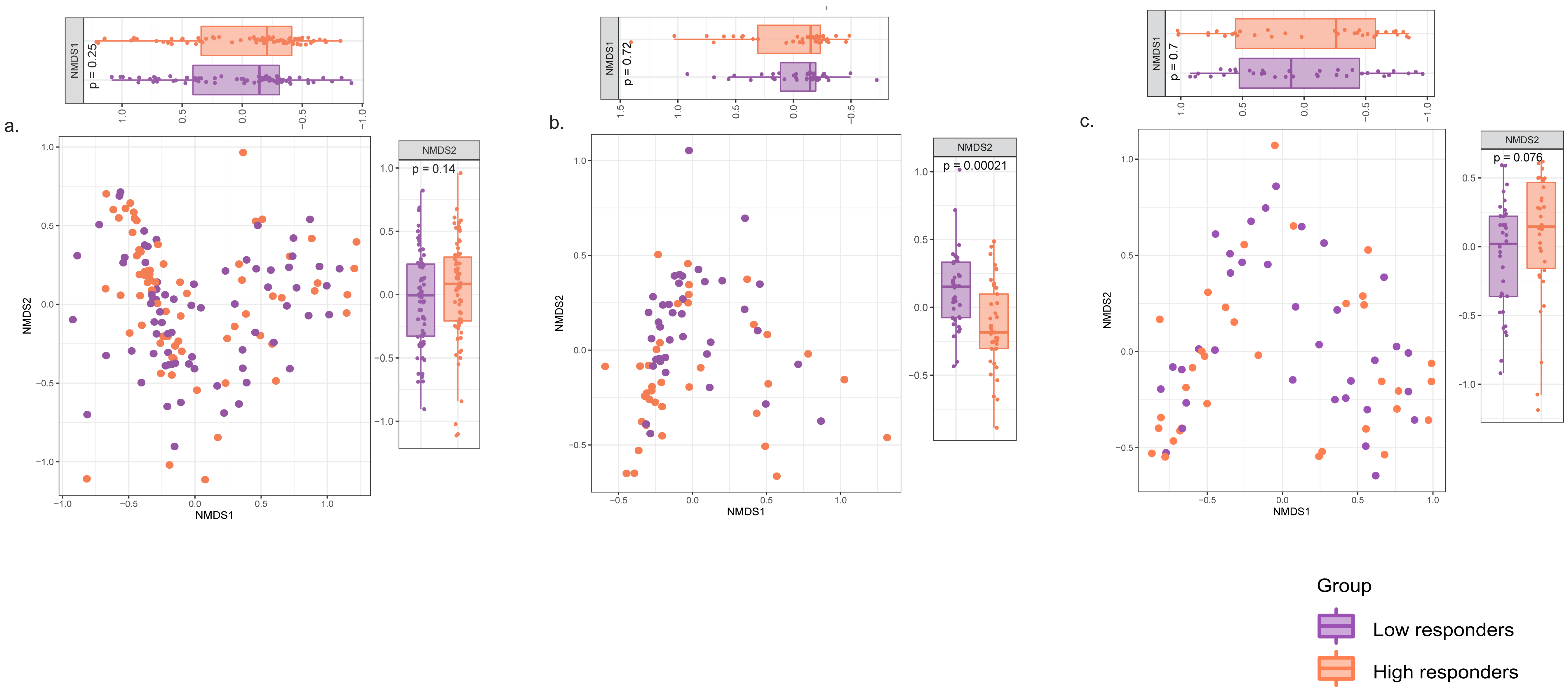

Supplementary Figure 2: β-diversity (NMDS) plots for investigating shifts in populations under high or responders for antibody levels in the (a) whole cohort, (b) HC group, and (c) PLWH group.

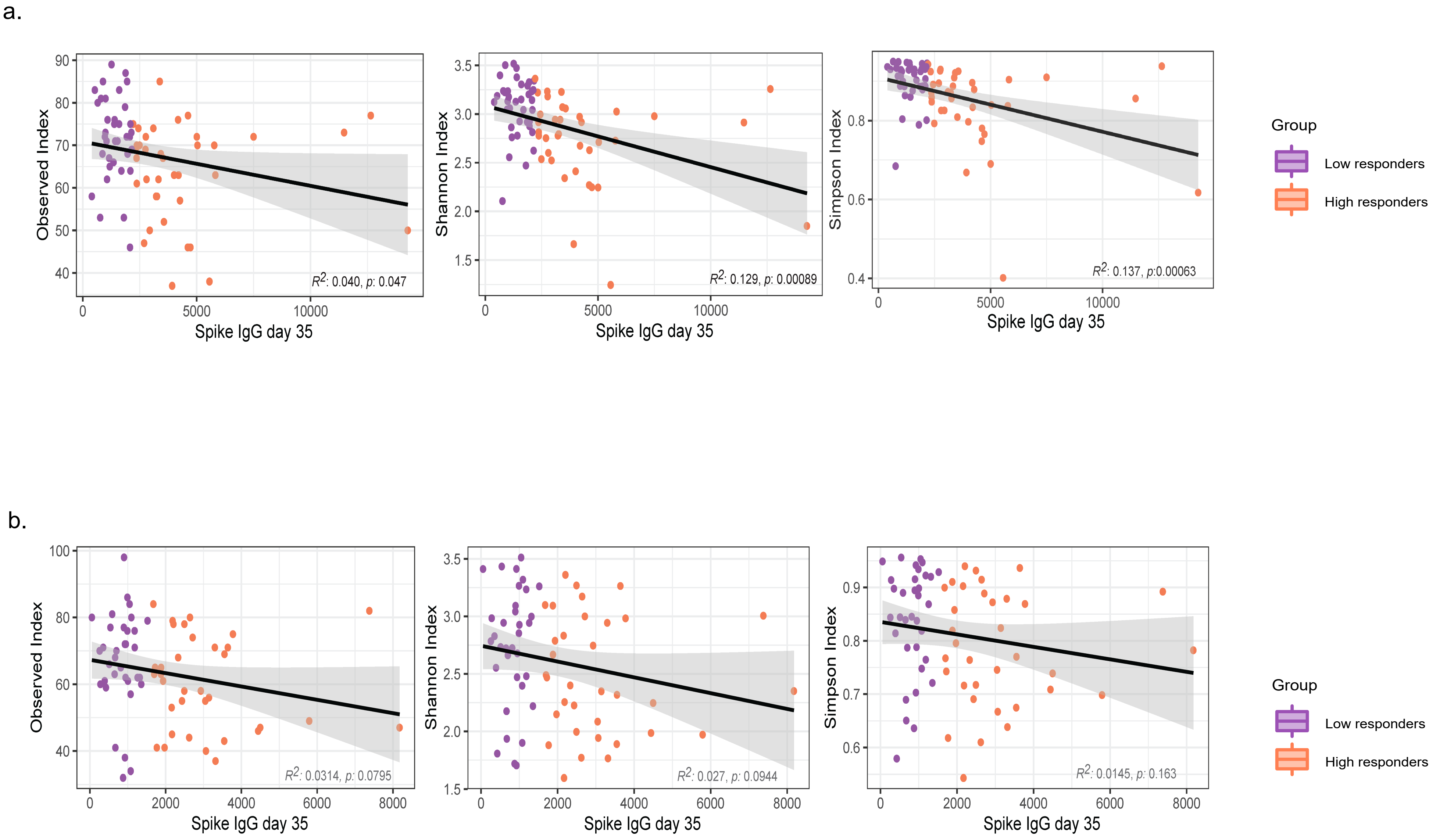

Supplementary Figure 3: Linear regression analysis between microbial diversity and antibody levels within the (a) HC group and within the (b) PLWH group.

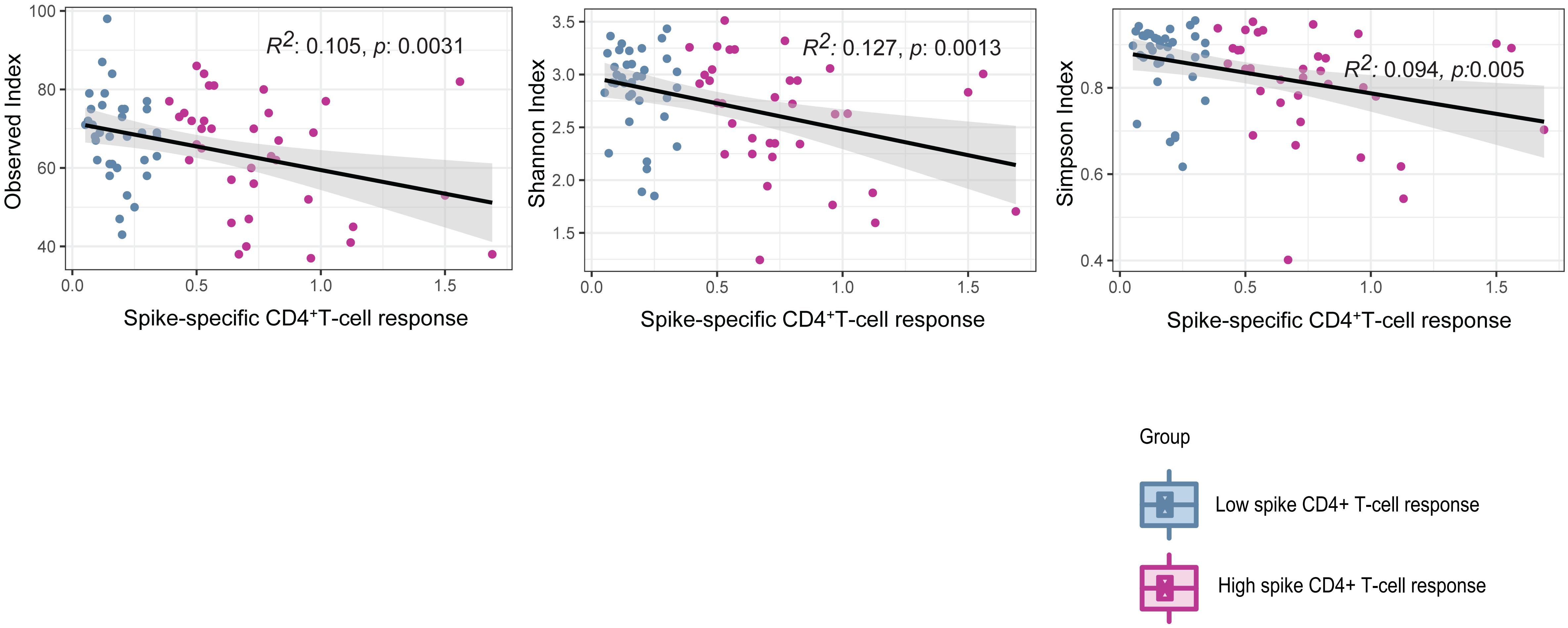

Supplementary Figure 4: Linear regression analysis between alpha diversity and spike-specific $CD4^+$ T-cell response in the whole cohort.

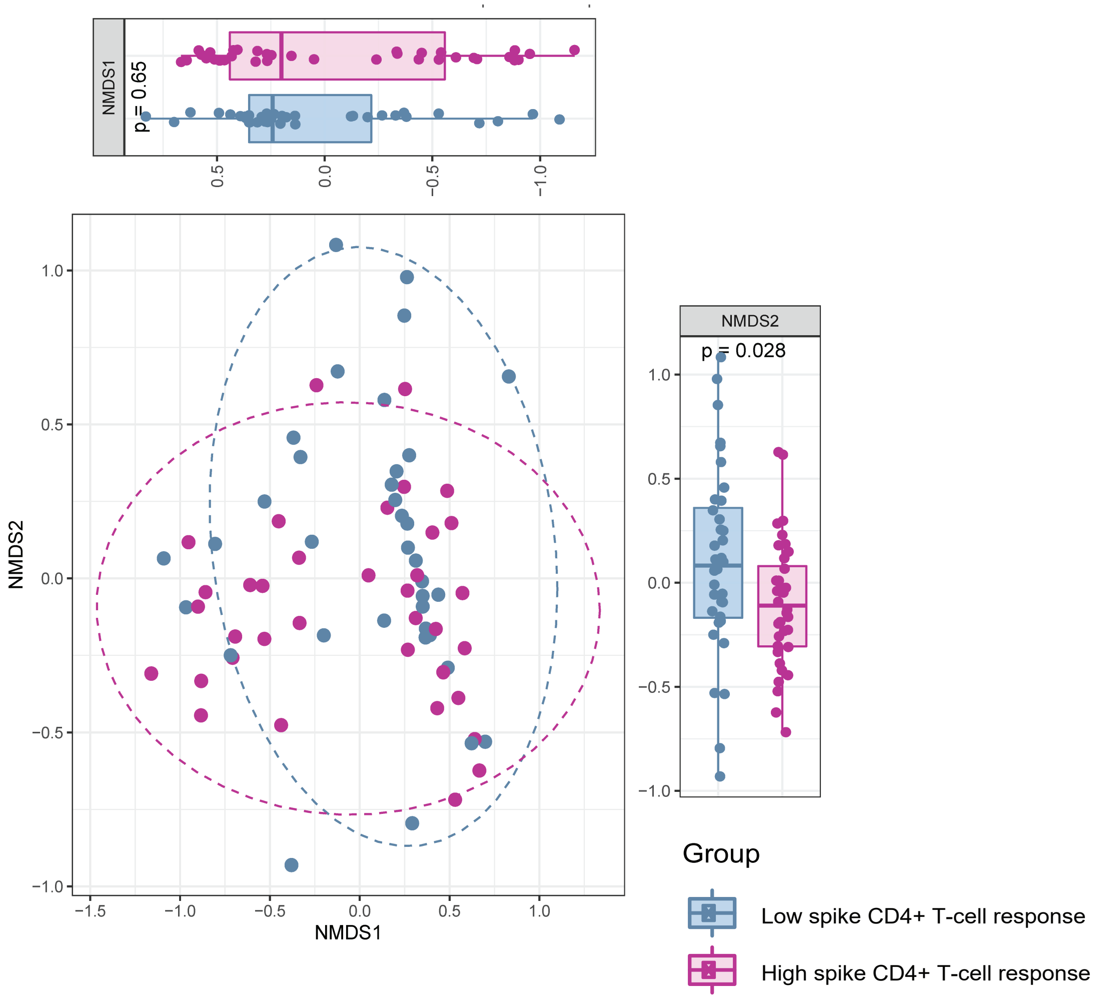

Supplementary Figure 5: β-diversity (NMDS) plots for two groups based on their spike-specific CD4⁺ T-cell responses in the whole cohort.

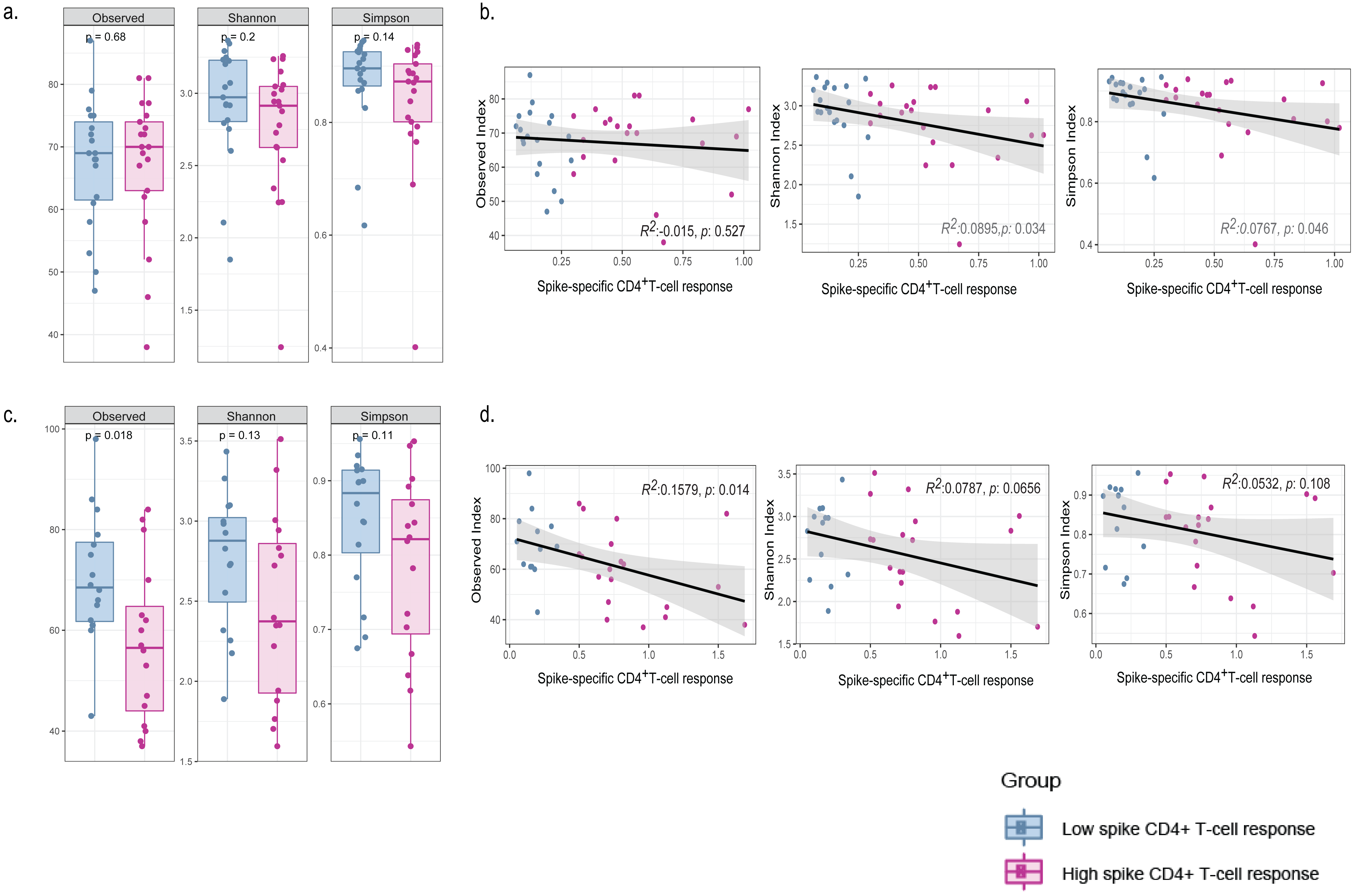

Supplementary Figure 6: Alpha diversity and linear regression analysis between individuals categorized by spike-specific $CD4^+$ T-cell responses within (a-b) HC and (c-d) PLWH.

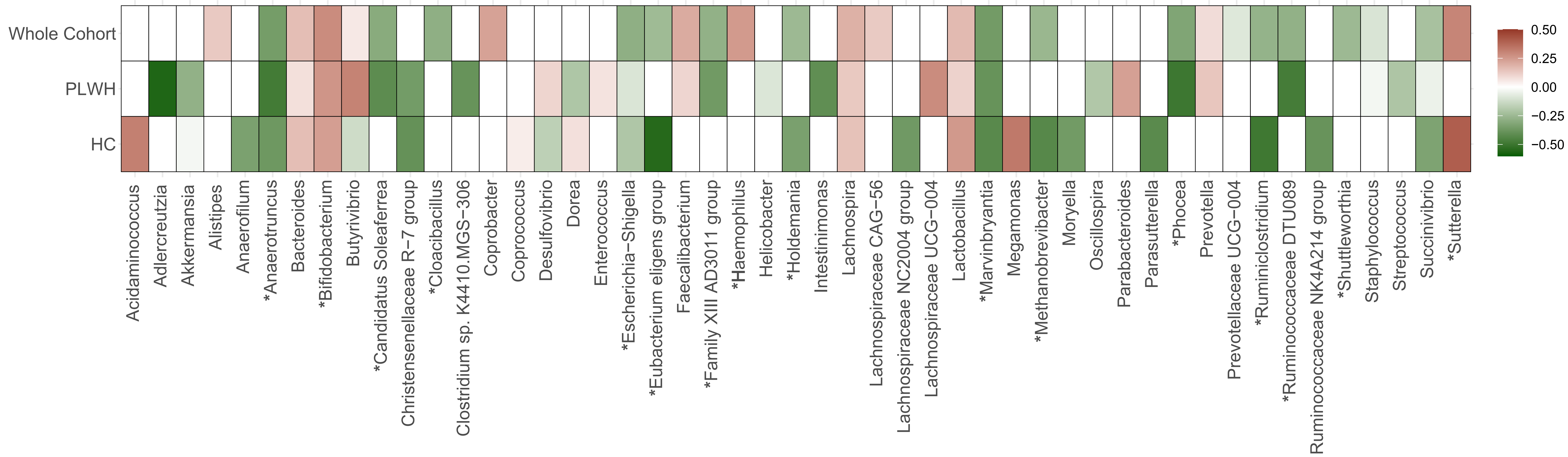

Supplementary Figure 7: Spearman correlation for associating the CD4+ T-cell responses-specific microbiome with antibody levels. The asterisks in the figure indicate the microbial taxa significantly correlated to spike IgG levels in the whole cohort. The microbes significantly correlated with antibody levels within HC and PLWH groups are outlined in supplementary table (Supplementary table 1).

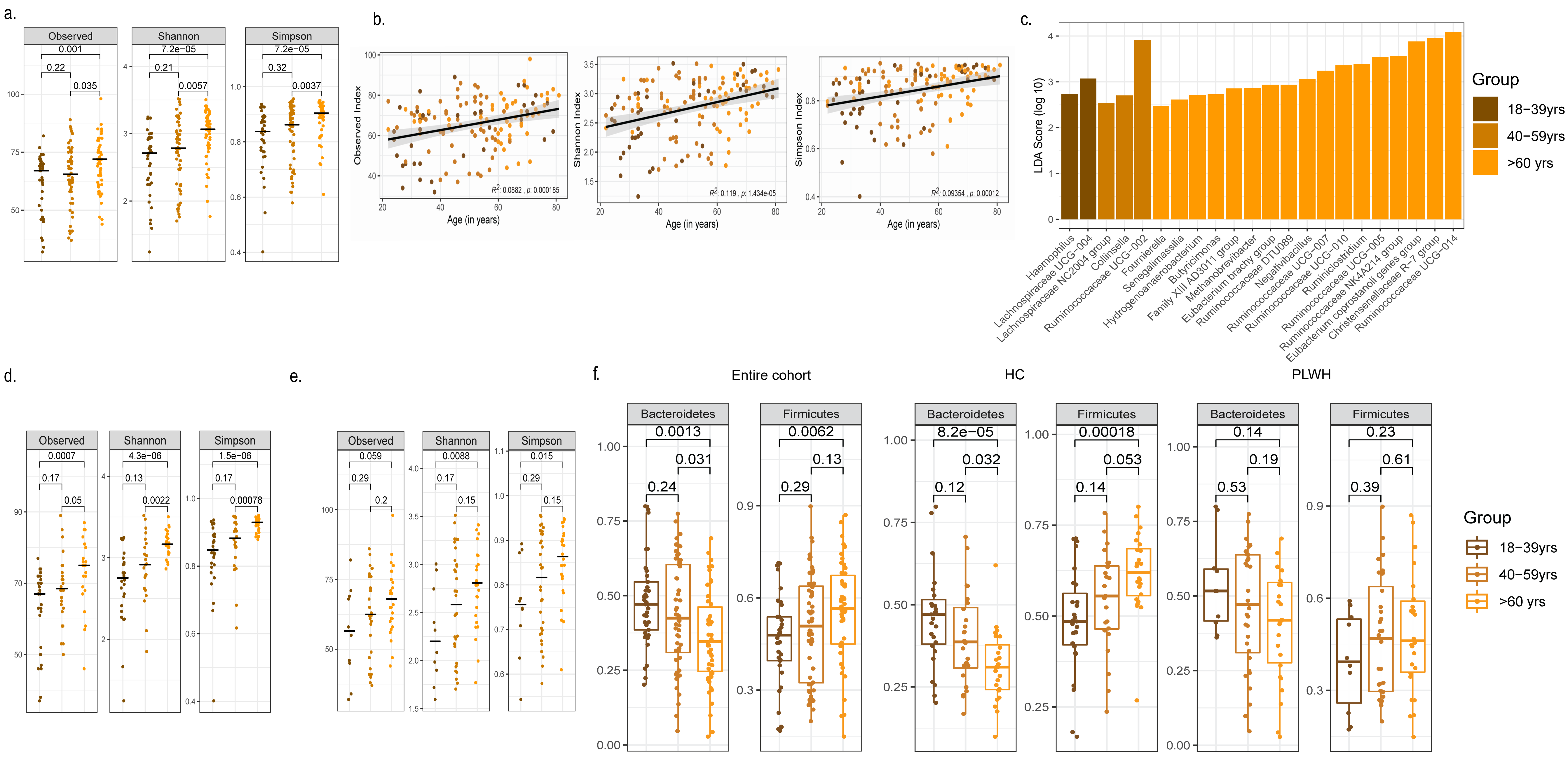

Supplementary Figure 8: Effect of age on gut microbiome. (a) Variation in the alpha diversity in the whole cohort among different age groups (18-39 years; n=37, 40-59 years; n=56 and >60 years; n=50). (b) Regression analysis showing the positive relationship between age and alpha diversity in the whole cohort among different age groups (18-39, 40-59 and >60 years). (c) Significantly abundant microbial markers for different age groups determined by LEfSe analysis with pairwise comparison. (d) Microbial diversity and richness in three different age groups within the HC group. (e) Bacterial diversity in three different age groups within the PLWH group. (f) Relative abundance of Firmicutes and Bacteroidetes in the whole cohort, HC and PLWH group. The Firmicutes: Bacteroidetes ratio is essential for gut balance.

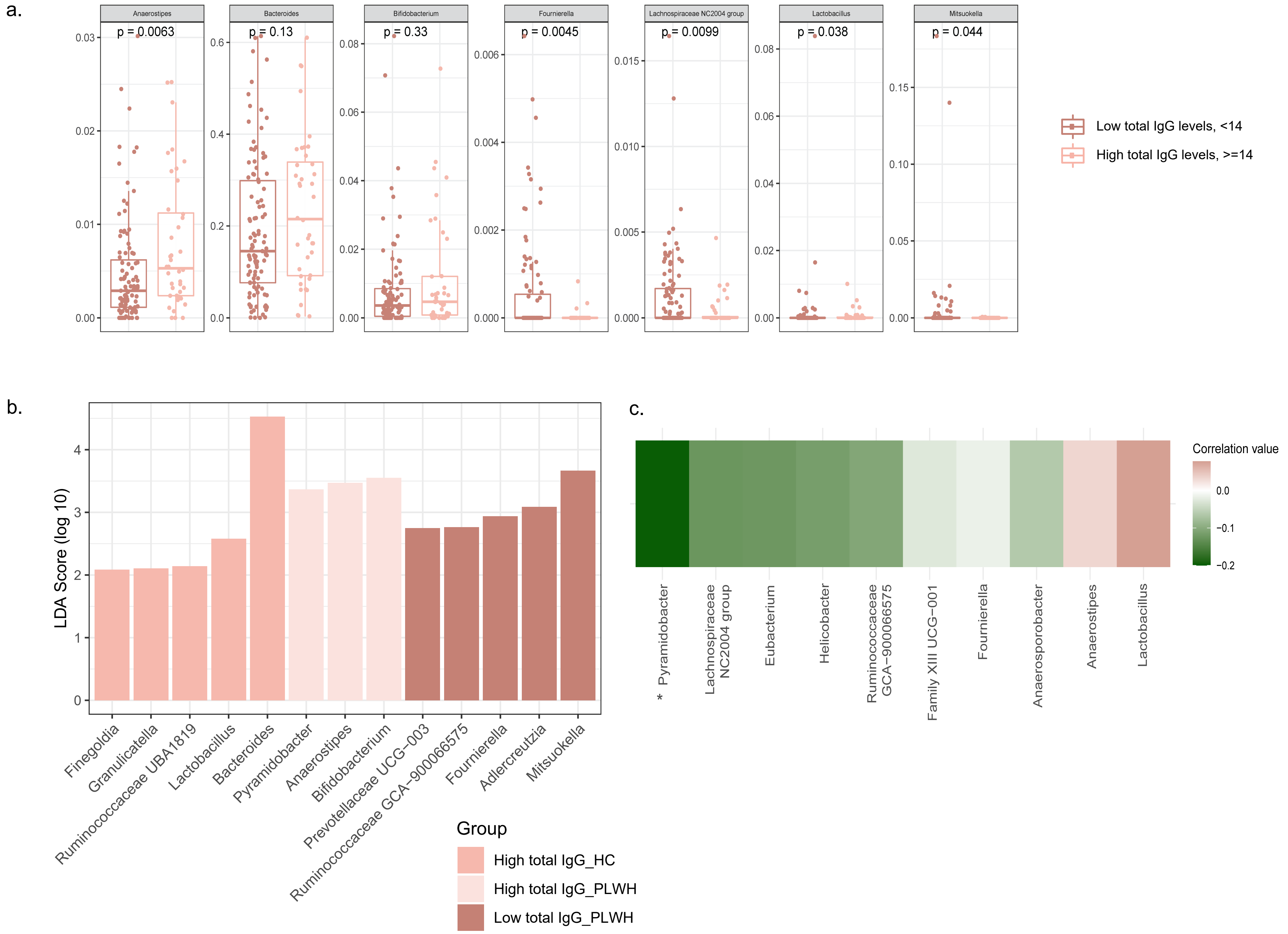

Supplementary Figure 9:(a) Changes in the abundance of microbial genus in individuals with different total IgG levels at baseline. (b) LDA analysis showing different significant bacterial taxa within the HC and PLWH group, with high and low total IgG levels at baseline. (c) Spearman correlation for associating baseline total IgG-specific microbiome with antibody levels.

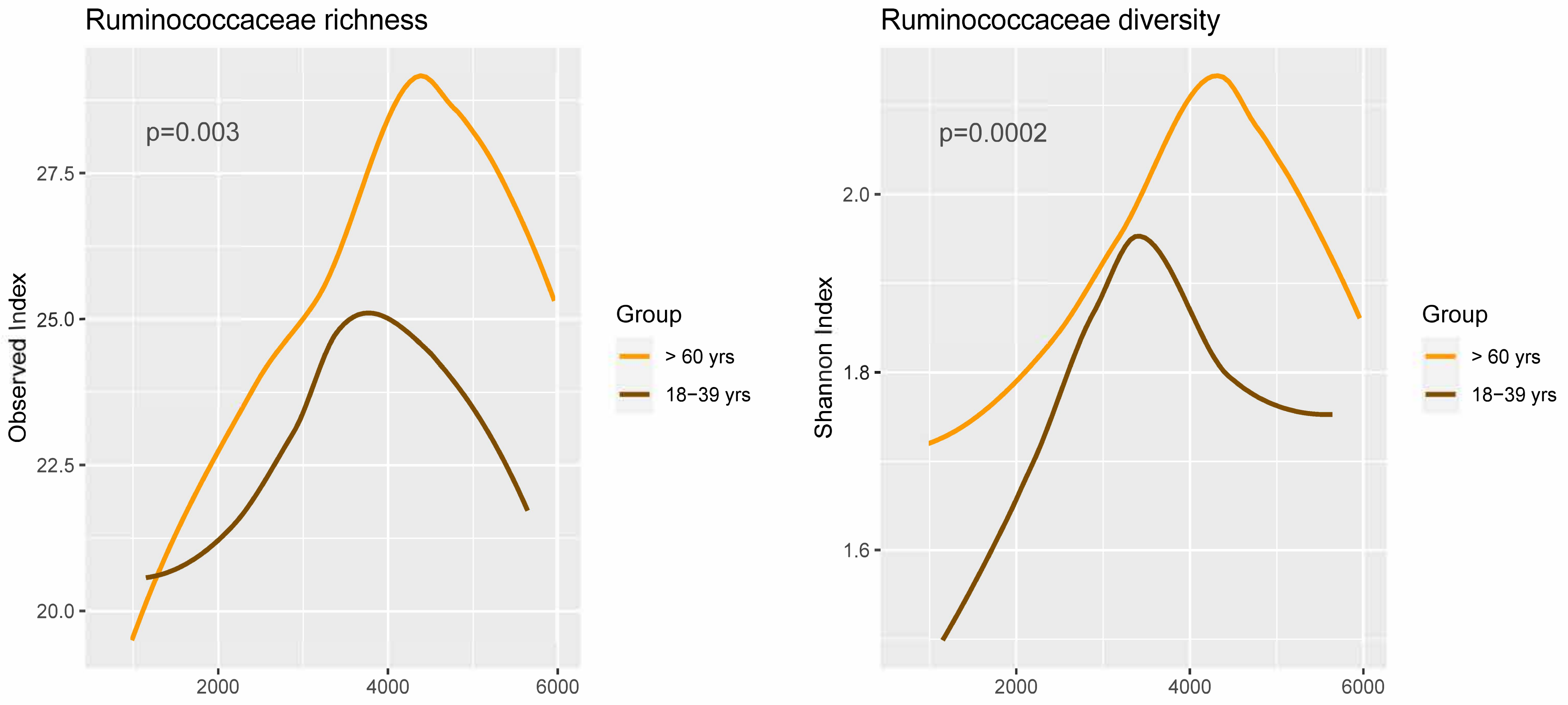

Supplementary Figure 10: Diversity and richness curve plot of Ruminococcaceae in different age groups (>60 years, n=50 and 18-39 years, n=37). P values were determined by paired Mann-Whitney U-test.
