# Supplementary material for: Impact of the gut microbiome on immunological responses to COVID-19 vaccination in healthy controls and people living with HIV
Source: NPJ Biofilms Microbiomes. 2023 Dec 20;9:104. doi: 10.1038/s41522-023-00461-w (PMC10733305; doi:10.1038/s41522-023-00461-w)
Supplement: Supplementary file 3 — Reporting Summary [file 41522_2023_461_MOESM3_ESM.pdf]

nature portfolio

Corresponding author(s): Shilpa Ray, NPJBIOFILMS-02417R

Last updated by author(s): Aug 11, 2023

Reporting Summary

Nature Portfolio wishes to improve the reproducibility of the work that we publish. This form provides structure for consistency and transparency in reporting. For further information on Nature Portfolio policies, see our Editorial Policies and the Editorial Policy Checklist.

Statistics

For all statistical analyses, confirm that the following items are present in the figure legend, table legend, main text, or Methods section.

n/a	Confirmed	
☐	☒	The exact sample size (*n*) for each experimental group/condition, given as a discrete number and unit of measurement
☐	☒	A statement on whether measurements were taken from distinct samples or whether the same sample was measured repeatedly
☐	☒	The statistical test(s) used AND whether they are one- or two-sided<br>*Only common tests should be described solely by name; describe more complex techniques in the Methods section.*
☐	☒	A description of all covariates tested
☐	☒	A description of any assumptions or corrections, such as tests of normality and adjustment for multiple comparisons
☐	☒	A full description of the statistical parameters including central tendency (e.g. means) or other basic estimates (e.g. regression coefficient) AND variation (e.g. standard deviation) or associated estimates of uncertainty (e.g. confidence intervals)
☐	☒	For null hypothesis testing, the test statistic (e.g. *F*, *t*, *r*) with confidence intervals, effect sizes, degrees of freedom and *P* value noted<br>*Give P values as exact values whenever suitable.*
☒	☐	For Bayesian analysis, information on the choice of priors and Markov chain Monte Carlo settings
☒	☐	For hierarchical and complex designs, identification of the appropriate level for tests and full reporting of outcomes
☐	☒	Estimates of effect sizes (e.g. Cohen's *d*, Pearson's *r*), indicating how they were calculated

Our web collection on statistics for biologists contains articles on many of the points above.

Software and code

Policy information about availability of computer code

Data collection	DNA was extracted from fecal samples of HC and PLWH group, and sent for 16S rRNA sequencing. Samples were sequenced on MiSeq (MSC 2.5.0.5/RTA 1.18.54) with a 301nt(Read1)-10nt(Index1)-10nt(Index2)-301nt(Read2) setup using 'Version3' chemistry. The Bcl to FastQ conversion was performed using bcl2fastq_v2.20.0.422 from the CASAVA software suite. The quality scale used is Sanger / phred33 / Illumina 1.8+
Data analysis	MultiQC v1.11, CutAdapt v1.18, Qiime2 v2019.10 (dada2), SILVA v132, R package ggplot2 v3.3.5, phyloseq v1.30.0, vegan R package v2.5.7, Deseq2 v1.26.0, Cytoscape v3.6.1, Python 2.7.17

For manuscripts utilizing custom algorithms or software that are central to the research but not yet described in published literature, software must be made available to editors and reviewers. We strongly encourage code deposition in a community repository (e.g. GitHub). See the Nature Portfolio guidelines for submitting code & software for further information.

Data

Policy information about availability of data

All manuscripts must include a data availability statement. This statement should provide the following information, where applicable:

- Accession codes, unique identifiers, or web links for publicly available datasets
- A description of any restrictions on data availability
- For clinical datasets or third party data, please ensure that the statement adheres to our policy

The metadata and raw 16S rRNA gene sequence data generated and analyzed during this study are deposited at the NCBI SRA database (Project number: PRJNA902956).

Research involving human participants, their data, or biological material

Policy information about studies with human participants or human data. See also policy information about sex, gender (identity/presentation), and sexual orientation and race, ethnicity and racism.

Reporting on sex and gender	Our findings do not apply to only one sex or gender. Sex and gender were considered during the study design and determined through self-reporting. Analyses on the data were done also independently of sex and gender, and the resulting data are shown and discussed in the manuscript. The clinical data obtained is not available for public sharing. We have performed sex- and gender-based analyses.
Reporting on race, ethnicity, or other socially relevant groupings	Our study was done on a cohort that was part of a bigger cohort selected for a clinical trial in another study (https://www.ncbi.nlm.nih.gov/pmc/articles/PMC8629680/). That study (and therefore our) targeted immunocompromised individuals among a Swedish population according only to their immunocompromised status. Ethnicity, however, has been reported in our study (self-reported).
Population characteristics	All population characteristics taken into account in this manuscript are mentioned in the demographic table and discussed in the manuscript. In brief, these include: Sex, age, BMI, Total IgG, lymphocytes and creatinine levels, ethnicity, diet, comorbidities, duration of antiretroviral treatment, CD4+ T cell count, CD4/CD8 ratio, and CD4 nadir.
Recruitment	This study's cohort was part of a bigger cohort from a previous study, a clinical trial (https://www.ncbi.nlm.nih.gov/pmc/articles/PMC8629680/). Recruitment for that study was done according to the immunocompromised status of the participants and approved by the Swedish Medical Product Agency (ID 5.1-2021-5881) and the Swedish Ethical Review Authority (ID 2021-00451).
Ethics oversight	Swedish Drug Agency- Läkemedelsverket EudraCT no. 2021-000175-37, clinicaltrials.gov no. 2021-000175-37; and Swedish Ethics Committee, Swedish Ethical Review Authority (ID 2021-00451)

Note that full information on the approval of the study protocol must also be provided in the manuscript.

Field-specific reporting

Please select the one below that is the best fit for your research. If you are not sure, read the appropriate sections before making your selection.

☒ Life sciences ☐ Behavioural & social sciences ☐ Ecological, evolutionary & environmental sciences

For a reference copy of the document with all sections, see nature.com/documents/nr-reporting-summary-flat.pdf

Life sciences study design

All studies must disclose on these points even when the disclosure is negative.

Sample size	The cohort used in this study is a subset of the cohort from a previous study (https://www.ncbi.nlm.nih.gov/pmc/articles/PMC8629680/). For that study, at the time of the study design, no information existed regarding the expected seroconversion rate of immunosuppressed individuals following vaccination with the mRNA BNT162b2 vaccine. Based on the initial BNT162b2 vaccine clinical trials results, we hypothesized that the proportion of seroconversion in healthy controls would be 99%. Choosing a sample size n=90 per group would give a power value of 81%, even with a conservatively low expected 10% difference in seroconversion in immunocompromised groups versus healthy controls. The final mPP group (n=468) represented a total reduction of approximately 10% of the study subjects.
Data exclusions	Subjects with detectable baseline spike antibodies against SARS-CoV-2, antibiotic treatment (three months before vaccination), and those with missing spike IgG data at day 35 were excluded from further analysis (PLWH: n=22; HC: n=15). All these exclusion criteria were predetermined and are a standard of microbiome studies, as they all result in variations of the microbiome composition.
Replication	Due to the specific nature of the premise of the study, the microbiome composition of individuals after two doses of Covid-19 vaccine, replication of this study is significantly hampered by the fact that a vast percentage of the global population has received 3 or more doses of said vaccine. Additionally, being a microbiome study, a different cohort might lead to different results, due to interpersonal microbiome variations.
Randomization	This is a study based on a non-randomized clinical trial (https://www.ncbi.nlm.nih.gov/pmc/articles/PMC8629680/) to assess the immune

Randomization	response to Covid-19 vaccination in immunocompromised individuals. Patients were selected according to their immunocompromised state and placed in the respective patient group. Both the diseased patient groups and the controls received the vaccine.
Blinding	During sample extraction, sequencing, and data acquisition, the IDs of the patients were not known to the individuals who were handling the respective processes.

Reporting for specific materials, systems and methods

We require information from authors about some types of materials, experimental systems and methods used in many studies. Here, indicate whether each material, system or method listed is relevant to your study. If you are not sure if a list item applies to your research, read the appropriate section before selecting a response.

Materials & experimental systems

n/a	Involved in the study
☒	☐ Antibodies
☒	☐ Eukaryotic cell lines
☒	☐ Palaeontology and archaeology
☒	☐ Animals and other organisms
☐	☒ Clinical data
☒	☐ Dual use research of concern
☒	☐ Plants

Methods

n/a	Involved in the study
☒	☐ ChIP-seq
☒	☐ Flow cytometry
☒	☐ MRI-based neuroimaging

Clinical data

Policy information about clinical studies

All manuscripts should comply with the ICMJE guidelines for publication of clinical research and a completed CONSORT checklist must be included with all submissions.

Clinical trial registration	EudraCT no. 2021-000175-37, clinicaltrials.gov no. 2021-000175-37
Study protocol	https://www.ncbi.nlm.nih.gov/pmc/articles/PMC8629680/
Data collection	The mRNA BNT162b2 (Comirnaty®, Pfizer/BioNTech) vaccine were assessed in immunocompromised patients and healthy controls at the Karolinska University Hospital, Stockholm, Sweden. The study started recruiting on Feb 15, 2021 and follow-up ended October 15, 2021.
Outcomes	The recent pandemic made us witness how significantly elderly and immunocompromised individuals were susceptible to coronavirus infection. PLWH, a group of immunocompromised individuals, are known to have poor immunogenicity to several oral and parenteral vaccines, including mRNA SARS-CoV-2 vaccines. It has also been established that PLWH have an altered microbiome during the course of the infection. This prompted us to investigate whether the altered microbiome is associated with the lower response to mRNA SARS-CoV-2 vaccination observed in PLWH. We therefore, collected the fecal samples from PLWH and healthy controls at baseline and sent them for 16S rRNA sequencing for further microbiome analysis.
